# Targeted Next Generation Sequencing for Human Papillomavirus Genotyping in Cervical Liquid-Based Cytology Samples

**DOI:** 10.3390/cancers14030652

**Published:** 2022-01-27

**Authors:** Karoline Andersen, Kasper Holm, Mette Tranberg, Cecilie Lebech Pedersen, Sara Bønløkke, Torben Steiniche, Berit Andersen, Magnus Stougaard

**Affiliations:** 1 Department of Clinical Medicine, Aarhus University, 8200 Aarhus N, Denmark; kaan@clin.au.dk (K.A.); kaho@clin.au.dk (K.H.); mettrani@rm.dk (M.T.); 201605240@post.au.dk (C.L.P.); torbstei@rm.dk (T.S.); berand@rm.dk (B.A.); 2 Department of Pathology, Aarhus University Hospital, 8200 Aarhus N, Denmark; sarasim@clin.au.dk; 3 Department of Public Health Programmes, Randers Regional Hospital, University Research Clinic for Cancer Screening, 8930 Randers NØ, Denmark

**Keywords:** next generation sequencing, human papillomavirus, HPV genotyping, cervical cancer screening, atypical squamous cells of undetermined significance

## Abstract

**Simple Summary:**

Testing for Human Papillomavirus (HPV) is currently being implemented as part of cervical cancer (CC) screening in several countries. However, infections with all but one of the HPV types classified as possibly carcinogenic cannot be detected by the assays used for CC screening today. The aim of our study was to demonstrate the use of a targeted next generation sequencing (NGS) HPV panel for CC screening—both general practitioner-collected samples and self-samples. We here show that the targeted HPV panel can detect HPV with a sensitivity and specificity similar to commercial HPV assays, one of which is used for CC screening today. However, the targeted HPV panel possess several advantages compared to the screening assays, as it enables specific detection of all relevant HPV types and can identify viral integration, variants in the HPV genome, and dominant HPV types in multi-infected cases.

**Abstract:**

At present, human papillomavirus (HPV) testing is replacing morphology-based cytology as the primary tool for cervical cancer screening in several countries. However, the HPV assays approved for screening lack detection for all but one of the possibly carcinogenic HPV types and do not genotype all included HPV types. This study demonstrates the use of a targeted HPV next generation sequencing (NGS) panel to detect and genotype all 25 carcinogenic, probably carcinogenic, and possibly carcinogenic HPV types as well as the low-risk types HPV6 and HPV11. The panel was validated using a cohort of 93 paired liquid-based cytology samples (general practitioner (GP)-collected cervical samples and cervico-vaginal self-samples (SS)). Overall, the targeted panel had a sensitivity (GP = 97.7%, SS = 92.1%) and specificity (GP = 98.0%, SS = 96.4%) similar to the commercial HPV assays, Cobas^®^ 4800 HPV DNA test (Roche) and CLART^®^ HPV4S assay (GENOMICA). Interestingly, of the samples that tested positive with the NGS panel, three (6.4%) of the GP-collected samples and four (9.1%) of the self-samples tested positive exclusively for HPV types only included in the NGS panel. Thus, targeted HPV sequencing has great potential to improve the HPV screening programs since, as shown here, it can identify additional HPV positive cases, cases with HPV integration, variants in the HPV genome, and which HPV type is dominant in multi-infected cases.

## 1. Introduction

Persistent infection with high-risk human papillomavirus (HPV) is the main cause of cervical cancer development [1]. Several HPV types are known to cause cervical cancer and, according to the International Agency for Research on Cancer (IARC), 12 of them are classified as carcinogenic to humans (Group 1) (HPV16, 18, 31, 33, 35, 39, 45, 51, 52, 56, 58, and 59) and 13 as probably (Group 2A) (HPV68) or possibly (Group 2B) (HPV26, 30, 34, 53, 66, 67, 69, 70, 73, 82, 85, and 97) carcinogenic to humans [2]. HPV is a non-enveloped double stranded DNA virus composed of six early genes (E1, E2, E4, E5, E6, and E7) encoding non-structural proteins and two late genes (L1 and L2) encoding structural proteins for construction of the viral capsid [3]. E6 and E7 are oncoproteins that target the p53 tumor suppressor protein and the retinoblastoma protein (pRB), respectively. This results in degradation of the targeted proteins, whereby the cell cycle is dysregulated causing uncontrolled cell division [4,5]. E2 has been shown to repress the expression of E6 and E7. However, the viral genome can be present either as an episomal infection or integrated into the host genome. In the last-mentioned case, E2 is frequently disrupted, resulting in upregulation of E6 and E7, which contributes to oncogenesis and the progression of cervical dysplasia to cervical cancer [6]. Studies have linked viral integration to increased risk of progression of cervical dysplasia to cervical cancer, implying the use of integration status as a potential biomarker for preneoplastic progression [7,8,9].

The causal link between persistent infection with high-risk HPV and cervical cancer has led to the development of multiple HPV detection assays [10]. HPV detection assays have shown a higher sensitivity for identifying cervical intraepithelial neoplasia grade 2 or worse (CIN2+) and have an improved negative predictive value compared to cytology [11,12]. Accordingly, Australia, England, and the Netherlands have implemented HPV testing as their primary cervical cancer screening tool [13,14,15]. This allows for longer screening intervals [14,16], which makes it increasingly important to avoid false negative screening results. Several countries, including Denmark, are currently implementing HPV testing in their cervical cancer screening program [17,18,19]. However, since most HPV infections are cleared spontaneously, risk stratification of HPV positive women becomes important to enable identification of women with a need for colposcopy referral [20,21]. Another challenge is that, even though most western countries have a well-implemented cervical cancer screening program, only approximately 60–80% of women invited to screening participates, despite several reminders to attend [22,23,24]. Therefore, to increase participation, a cervico-vaginal sample taken at home (a self-sample) for HPV testing has been proposed as an alternative for non-attenders [25,26,27,28,29].

Traditionally, PCR-based amplification has been used for HPV detection with most tests detecting the viral capsid protein L1, including the Cobas^®^ 4800 HPV DNA test (Roche, Hvidovre, Denmark) and the CLART^®^ HPV4S assay (GENOMICA, Madrid, Spain) [30]. Former studies have shown high sensitivity and specificity for HPV detection using next generation sequencing (NGS), which enables detection of multiple targets simultaneously [31]. Previously, a study from our group demonstrated the use of a small targeted NGS panel for HPV detection and genotyping in both formalin-fixed, paraffin-embedded (FFPE) samples and plasma samples. Furthermore, the study demonstrated the use of the targeted NGS panel for detection of viral integration of HPV16 [32].

In this study, the small targeted NGS panel has been further optimized, and we aimed to examine the feasibility of the NGS assay for HPV detection and genotyping in liquid-based cytology (LBC) samples (general practitioner (GP)-collected cervical samples and cervico-vaginal self-samples). Furthermore, we aimed to examine if the designed NGS panel can assess the viral integration status as well as detect variants in the HPV genome.

## 2. Materials and Methods

### 2.1. Study Cohort

The study cohort was extracted from a cohort of 213 women aged 30–59 years, diagnosed with atypical squamous cells of undetermined significance (ASC-US), and reflex HPV tested within the Danish cervical cancer screening program between June 2015 and December 2016 [33,34]. From the original cohort, 47 Cobas^®^ 4800 HPV positive and 46 Cobas^®^ 4800 HPV negative GP-collected cervical samples with paired cervico-vaginal self-samples collected at home were selected (median no. of days between samples: 43, range: 13–95 days). Both GP-collected samples and self-samples from each woman in the study cohort had been analyzed with Cobas^®^ 4800 (Roche, Hvidovre, Denmark) and CLART^®^ HPV4S (GENOMICA, Madrid, Spain) for use in previous publications, and the recruitment procedure as well as the sample collection, processing, storage, and analysis have been described elsewhere [33,34].

### 2.2. NGS Panel Design

The Ion Torrent NGS panel was designed using the AmpliSeq Designer (Thermo Fisher Scientific, Roskilde, Denmark) and included the 25 IARC-classified carcinogenic (HPV16, 18, 31, 33, 35, 39, 45, 51, 52, 56, 58, 59), probably carcinogenic (HPV68), and possibly carcinogenic (HPV26, 30, 34, 53, 66, 67, 69, 70, 73, 82, 85, 97) HPV types. Furthermore, the panel included the two most common low-risk HPV types HPV6 and HPV11 [2]. Cobas^®^ 4800 can individually genotype HPV16 and HPV18 and can detect HPV31, 33, 35, 39, 45, 51, 52, 56, 58, 59, 66, and 68 as a pooled result, where the specific HPV type is not specified. CLART^®^ HPV4S can individually genotype all HPV types included in Cobas^®^ 4800 and can, in addition, genotype the low-risk HPV types HPV6 and HPV11.

For each of the 27 HPV types included in the NGS panel, eight amplicons were designed. Two amplicons were designed for each of the E6 and E7 oncogenes and, for comparison with the L1-detecting analyses such as Cobas^®^ 4800 and CLART HPV4S, one amplicon was designed for L1. Additionally, three amplicons were designed for E2, whereof two were in the E2 region called the hinge-region [35]. E2, and particularly the hinge-region, was covered by amplicons, since this is a hotspot for integration of HPV into the host genome resulting in the deletion of this part of the viral genome [6,36,37].

To confirm the presence of DNA in each sample, an amplicon for each of five human reference genes (BTF3, PABPN1, PPIE, RAB1B, SRSF3) were included in the panel as well. How to select appropriate human reference genes is described in a previous publication [32]. The panel was designed with short amplicons of approximately 90 base pairs using the circulating free DNA (cfDNA) pipeline of the AmpliSeq Designer.

The NGS panel was validated using DNA from paired GP-collected cervical samples and cervico-vaginal self-samples. The DNA had been purified for a previous study using the Cobas x480 Instrument (Roche, Hvidovre, Denmark) and analyzed using the Cobas^®^ 4800 and CLART^®^ HPV4S assays [33,34].

### 2.3. Preparation of Samples and Sequencing

Excess DNA, purified for Cobas^®^ 4800 as per routine in the cervical cancer screening program, was used for the NGS assay. The samples were prepared for sequencing using the Ion AmpliSeq Library Kit 2.0 (Thermo Fisher Scientific, Roskilde, Denmark) according to the manufacturer’s protocol, except that the reactions were performed in half volume. Input DNA was in the range of 10–60 ng for the self-samples. Due to limited material, the input DNA concentration was not measured for the GP-collected samples. However, the DNA concentrations were measured for six comparable GP-collected samples, not used for NGS, from the original cohort of 213 women. The DNA concentrations of these samples were very low (mean: 2.42 ng/µL, range: 0.38–6.95 ng/µL), indicating very low DNA concentrations of the GP-collected samples included in this study as well. Therefore, 6 µL of undiluted DNA was used for library preparation of the GP-collected samples. The GP-collected samples were labelled with Ion Xpress Barcode Adapters (Thermo Fisher Scientific, Roskilde, Denmark), but in an attempt to reduce the amount of background, the self-samples were labelled with Ion Torrent Dual Barcodes (Thermo Fisher Scientific, Roskilde, Denmark). Before pooling the libraries for template preparation and sequencing, the libraries were quantified using the Ion Library TaqMan Quantitation Kit (Thermo Fisher Scientific, Roskilde, Denmark) and diluted to 50 pM. Template preparation was performed using the Ion 510 & Ion 520 & Ion 530 Kit—Chef (Thermo Fisher Scientific, Roskilde, Denmark) on the Ion Chef System (Thermo Fisher Scientific, Roskilde, Denmark). Sequencing was performed using the Ion GeneStudio S5 System (Thermo Fisher Scientific, Roskilde, Denmark) with the coverage analysis plugin set to a minimum amplicon length of 50 base pairs.

### 2.4. NGS Data Analysis

The sequencing data from each sample were aligned to a custom reference sequence composed of the human reference genome (hg19) and the genomes of 286 HPV types. The applied HPV reference genomes have been used in a former study [32], where a complete list of the accession numbers can be found. The data, generated by the S5 Torrent Server (Thermo Fisher Scientific), were delivered as a coverage analysis Excel file containing the total number of reads for each amplicon, including both HPV amplicons and amplicons of the five human reference genes for all samples. The inclusion of the five human reference genes were to ensure that an HPV negative sample was indeed negative and not the result of a flawed analysis; thus, all five genes should be amplified and sequenced for an HPV negative sample to be valid. Based on the reads of the E6 and E7 HPV16 amplicons, a cut-off was calculated. Since HPV16 was the most prevalent HPV type and the HPV type with the highest number of reads, the cut-off was based on this type, creating the most background reads. Since both E2 and L1 have previously been reported to be deleted following integration [6,32,38,39], the cut-off was based solely on the E6 and E7 amplicons. The amount of background of the E6 and E7 HPV16 amplicons was assessed using the samples that had tested HPV16 negative in both the Cobas^®^ 4800 and CLART^®^ HPV4S assays, since the HPV16 reads present in these samples would correspond to background reads. The cut-off was defined as the mean of the HPV16 E6 and E7 amplicons’ limit of detection (LoD). The LoD for each HPV16 E6 and E7 amplicon was calculated as follows: $LoD=mean_{blank}+\left(2*SD_{blank}\right)$. To render a woman HPV positive for a given HPV type, the number of reads for all four E6 and E7 amplicons had to be above the cut-off. However, if the number of reads for three out of four of the E6 and E7 amplicons were high (>500 reads), the woman was rendered HPV positive as well. Poor quality amplicons, i.e., amplicons with read depth of <1% of the total amount of reads on the specific HPV type in at least 25% of the HPV cases positive for that given HPV type, were not included when applying the cut-off to different HPV types.

### 2.5. Statistics

The NGS, Cobas^®^ 4800, and CLART^®^ HPV4S assays were compared regarding sensitivity and specificity for HPV detection in general as well as for HPV16 detection. Each assay was compared to the two other assays to assess a “true/consensus” HPV result, which means that when at least two of the assays showed the same HPV result, this result was interpreted to be correct. For example, if the NGS assay did not detect any HPV types/HPV16, but the two other assays detected HPV/HPV16, the result of the NGS assay was interpreted as being false negative. The three assays were compared only for the HPV types detectable with all three HPV assays (HPV16, 18, 31, 33, 35, 39, 45, 51, 52, 56, 58, 59, 66, and 68). Sensitivity, specificity, and corresponding 95% confidence intervals (CI) were calculated for each of the three HPV assays for both GP-collected samples and self-samples.

The specific HPV types identified by the NGS assay in the study cohort were compared to the HPV types identified by CLART^®^ HPV4S. The Cobas^®^ 4800 assay was excluded from this comparison, as this assay only genotypes HPV16 and HPV18. The NGS and CLART^®^ HPV4S assays were compared only for the HPV types detectable with both HPV assays (HPV6, 11, 16, 18, 31, 33, 35, 39, 45, 51, 52, 56, 58, 59, 66, and 68). The degree of agreement between the NGS assay and the CLART^®^ HPV4S assay was categorized as follows: Agreement was defined as detection of the exact same HPV types in the NGS assay and in CLART^®^ HPV4S. Partial agreement was applied in samples infected with multiple HPV types and defined as detection of at least one of the same HPV types in the NGS assay and CLART^®^ HPV4S. Disagreement was defined as detection of none of the same HPV types between the NGS assay and CLART^®^ HPV4S. The percentage of agreement between NGS and CLART^®^ HPV4S was calculated as the number of samples with agreement divided by the total number of samples.

### 2.6. Ethical Approval

According to the EUs General Data Protection Regulation, the project was listed at the record of processing activities for research projects in the CDR (journal No.: 1-10-72-69-15). The study was approved by the local Ethical Committee of the Central Denmark Region (journal No.: 1-16-02-209-15).

## 3. Results

### 3.1. HPV Genotyping

In total, 93 paired GP-collected samples and self-samples were genotyped using the NGS assay. All human reference genes were amplified, and the mean number of reads were 28,425 (range: 13,454–49,093) for the HPV negative GP-collected samples and 28,242 (range: 8544–49,823) for the HPV negative self-samples, confirming the presence of suitable quality DNA material. Using the NGS assay, a total of 15 women had discordant results. Nine women (9.7%) were GP-collected HPV positive samples/self-sample HPV negative samples. Six women (6.5%) were self-sample HPV positive samples/GP-collected HPV negative samples. Several different HPV types were identified in the study cohort: HPV6, 16, 18, 30, 31, 33, 35, 39, 45, 51, 52, 53, 56, 58, 59, 66, 67, 68, 69, 70, 73, and 82 (Table 1). Twenty-five of the HPV positive GP-collected samples (53.2%) and 24 of the HPV positive self-samples (54.5%) showed infection with multiple HPV types. The HPV types detected for each woman are presented in Table S1.

Several possibly carcinogenic HPV types only included in the NGS assay were identified (HPV30, 53, 67, 69, 70, 73, and 82) (Table S1). One or more of these types were detected in 16 (34.0%) of the HPV positive GP-collected samples and in 18 (40.9%) of the HPV positive self-samples. Interestingly, out of the samples that tested positive with the NGS assay, three (6.4%) of the GP-collected samples and four (9.1%) of the self-samples tested positive exclusively for HPV types only included in the NGS assay (HPV30, 53, 67, and 70). Thus, these were not detectable with the Cobas^®^ 4800 or CLART^®^ HPV4S assays (patient No. 64, 92, and 211 for the GP-collected samples and patient No. 21, 107, 130, and 211 for the self-samples). However, two of the three mentioned GP-collected samples had a Cobas^®^ 4800 HPV positive result anyway. For these two samples, one of them tested HPV16 positive using Cobas^®^ 4800, but since HPV16 was not detected neither by any other assay nor by Cobas^®^ 4800 itself in the self-sample; this is most likely a false positive result. The other sample tested positive for an unspecified HPV type using Cobas^®^ 4800. Since the self-sample from this patient tested HPV positive using the three different HPV assays, it is difficult to conclude whether this is a false positive sample or not. However, Cobas^®^ 4800 has been observed to cross-react with HPV types not included in the assay [40]. This includes cross-reactivity for HPV70, which is present in both of the two mentioned samples when tested with NGS.

### 3.2. Sensitivity and Specificity for HPV Detection

For the GP-collected samples, the sensitivity of the NGS, Cobas^®^ 4800, and CLART^®^ HPV4S assays for HPV detection was 97.7% (95% CI: 87.7–99.9%), 93.0% (95% CI: 80.9–98.5%), and 88.4% (95% CI: 74.9–96.1%), respectively. The specificity of the same samples using the three assays was 98.0% (95% CI: 89.4–100.0%), 86.0% (95% CI: 73.3–94.2%), and 98.0% (95% CI: 89.4–100.0%), respectively. For the self-samples, the sensitivity was 92.1% (95% CI: 78.6–98.3%), 94.7% (95% CI: 82.3–99.4%), and 97.4% (95% CI: 86.2–99.9%), respectively, and the specificity of the same samples was 96.4% (95% CI: 87.5–99.6%), 90.9% (95% CI: 80.1–97.0%), and 100.0% (95% CI: 93.5–100.0%), respectively (Table 2).

### 3.3. Concordance in HPV Genotyping

The specific HPV types genotyped using the NGS assay were compared to the CLART^®^ HPV4S genotyping results. Among the GP-collected samples, 75 (80.7%) were categorized as agreement, ten (10.8%) as partial agreement, and eight (8.6%) as disagreement. For the self-samples, 78 (83.9%) showed agreement, eight (8.6%) showed partial agreement, and seven (7.5%) showed disagreement (Table 3). Two of the disagreement cases in the GP-collected samples were caused by detection of HPV only when using CLART^®^ HPV4S while the other six were caused by HPV detected only when using the NGS assay. The Cobas^®^ 4800 assay agreed with the NGS assay in detection/no detection of HPV in six of the disagreement cases and with the CLART^®^ HPV4S assay in the two other disagreement cases. For the self-samples showing disagreement, HPV was detected solely by CLART^®^ HPV4S in three samples and solely by NGS in the other four samples showing disagreement. Here, the Cobas^®^ 4800 assay agreed with the NGS assay in detection/no detection of HPV in one case and with the CLART^®^ HPV4S assay in five of the disagreement cases. The last disagreement case in the self-samples showed detection of HPV6 using NGS. Since this HPV type is not included in the Cobas^®^ 4800 assay, Cobas^®^ 4800 could not be used for comparison in this case. The detected HPV types for each assay are presented in Table S1 for both GP-collected samples and self-samples.

### 3.4. Sensitivity and Specificity for HPV16 Detection

Since HPV16 is genotyped by all three assays, the sensitivity and specificity for detection of HPV16 were assessed as well. Moreover, HPV16 is the most frequent HPV type and accounts for approximately 60% of cervical cancers [41]. Due to few HPV16 positive samples in the relatively small study cohort, the GP-collected samples and self-samples were pooled. The samples were independent of each other, taken on different days and from different areas of the cervix and vagina. The sensitivity of HPV16 detection in the NGS, Cobas^®^ 4800, and CLART^®^ HPV4S assays was 100.0% (95% CI: 81.5–100.0%), 100.0% (95% CI: 81.5–100.0%), and 83.3% (95% CI: 58.6–96.4%), respectively. The specificity of HPV16 detection using the same assays was 98.8% (95% CI: 95.8–99.9%), 95.8% (95% CI: 91.6–98.3%), 100.0% (95% CI: 97.8–100.0%), respectively (Table 4).

### 3.5. Assessment of the Viral Integration State

A former study published by our group showed the ability to detect an episomal as well as an integrated HPV16 viral state [32]. As described earlier, integration of HPV into the host genome often occurs in the E2 hinge-region, and therefore, in the NGS panel designed for this study, E2 amplicons were designed for each of the 27 included HPV types. Since the absence of E2 amplicons indicates HPV integration, this enables an assessment of whether the sample has episomal or integrated HPV. The HPV18 positive patient no. 96 showed a clear integration pattern with no reads in any of the three included E2 amplicons of HPV18 as depicted in Table 5. The patient no. 96 depicted in Table 5 was further positive for a non-integrated HPV31. The lower number of reads in the self-sample (Table 5) was a trend we saw for the vast majority of paired samples and might be due to the general practitioner sampling more precisely from the cervix compared to self-sampling. This illustrates the NGS assay’s ability to detect viral integration also in HPV types different from HPV16. However, this panel is designed with the main purpose of HPV genotyping, thus only containing few regions of each HPV type to ensure high coverage at low cost. Since studies have shown that integration can cause breakpoints at various sites of the HPV genome not covered in this NGS panel [6,38,42], a panel with the main purpose of assessment of the viral integration state should contain additional regions of each HPV type.

### 3.6. Detection of HPV Variants

When a variant caller was applied to the data, the NGS assay detected multiple genomic variants of the different HPV types included (Table S2). The number of variants detected for each HPV type ranged from one (HPV35) to 13 (HPV66) variants. By detection of different HPV variants, determination of HPV sub-lineages may be possible. However, this NGS panel was not designed for determination of HPV sub-lineages, and thus, the included regions of each HPV type do not necessarily contain the genomic positions for determination of sub-lineages of the identified HPV types.

## 4. Discussion

Our NGS assay is a semi-quantitative method that can detect and genotype HPV and includes all 25 carcinogenic, probably carcinogenic, and possibly carcinogenic HPV types as well as the low-risk HPV types HPV6 and HPV11. When comparing the sensitivity and specificity of HPV detection using the NGS, Cobas^®^ 4800, and CLART^®^ HPV4S assays, the three assays showed a similar performance both when examining GP-collected samples and self-samples. The same was observed for specific detection of HPV16 when examining both sample types. The specific HPV types genotyped with NGS showed good concordance with the CLART^®^ HPV4Sassay, where agreement was obtained in 80.7% of GP-collected samples and 83.9% of self-samples. Furthermore, our NGS assay detected viral integration in an HPV18 positive sample and multiple genomic variants of the different detected HPV types. Interestingly, of the samples that tested positive with the NGS assay, three (6.4%) of the GP-collected samples and four (9.1%) of the self-samples tested positive exclusively for HPV types only included in the NGS assay (HPV30, 53, 67, and 70).

Since multiple studies have shown carcinogenicity of the above-mentioned HPV types HPV53, 67, and 70 as well as the HPV types HPV26, 73, and 82 [43,44,45] whereof all are classified as possibly carcinogenic, inclusion of these HPV types in HPV assays approved for screening should be considered. Especially since several countries are currently changing their cervical cancer screening programs to primary screening for HPV rather than cytological detection of cervical dysplasia [13,14,17,18]. Additionally, with the introduction of HPV testing as the primary screening tool, a change in the screening interval from every 3rd year to every 5th or 10th year has been proposed [14,16]. This is because HPV testing is believed to increase the sensitivity of high-grade cervical intraepithelial neoplasia detection in screening [12,16]. Thus, it becomes important to minimize the number of false negatives. However, the HPV assays approved by the U.S. Food and Drug Administration (FDA) only cover 13–14 HPV types and thus all lack detection of 11–12 HPV types classified as possibly carcinogenic. This includes the HPV types HPV30, 53, 67, and 70, which in our study cohort were detected in some patients without detection of any of the HPV types included in screening assays today. Furthermore, the FDA-approved assays are not able to distinguish all detectable HPV types from each other, i.e., the assays cannot genotype all included HPV types [46]. The lack of possibly carcinogenic HPV types and HPV genotyping in the FDA-approved assays makes it difficult to differentiate between women with same genotype persistence and genotype switch. Differentiation of these two groups of women is important, since only persistent HPV infections can develop to cervical cancer [1,47], and this is enabled by our NGS assay with the inclusion and genotyping of all carcinogenic, probably carcinogenic, and possibly carcinogenic HPV types.

Since an increase of colposcopy referrals have been observed with the shift to HPV detection in cervical cancer screening [13,48], an introduction of all possibly carcinogenic HPV types into cervical cancer screening could potentially result in an additional increase of colposcopy referrals. The reason for this is that most HPV infections clear spontaneously without the need of any treatment. The dilemma of detecting and preventing more cervical cancer cases at the expense of increased colposcopy referrals could potentially be solved by better risk stratification of HPV positive women. Our NGS assay can potentially help solve this problem with its ability to genotype the 25 carcinogenic, probably carcinogenic, and possibly carcinogenic HPV types and, in addition, assess the viral integration state and detect viral variants while being semi-quantitative. Since the NGS assay is semi-quantitative, it gives an indication of which HPV type is dominant in women with multiple HPV infections and can, moreover, be used as a measure of the viral load in cervical biopsies. Viral load is influenced by factors such as sampling, age, infection with multiple HPV types, and area of cervical lesion [49] but a high viral load has been related to high-grade cervical dysplasia [7,8]. The same has been observed for the viral integration status where viral integration has been linked to increased risk of progression of cervical dysplasia to cervical cancer [7,8,9]. Moreover, HPV types can be classified into lineages (that diverge by 1–10% in sequence) and sub-lineages (that diverge by 0.5–1% in sequence) [50] and specific HPV sub-lineages have been linked to persistent infection and increased risk of cervical cancer development as well [51,52,53,54]. Further, the risk of precancer/cancer for specific sub-lineages have been reported to depend on the women’s race/ethnicity where a race/ethnicity matching that of the infecting HPV16 sub-lineage increased the risk of CIN3+ [51]. With the NGS assay’s ability to detect viral variants, amplicons could be designed to cover specific areas that would make sub-lineage classification possible. Lastly, some specific HPV variants have been related to persistent infection and increased risk of progression to high-grade cervical dysplasia or cervical cancer as well [53,54] and these specific variants could, therefore, also be covered by our NGS assay. Thus, the NGS assay performs as well as Cobas^®^ 4800 and CLART^®^ HPV4S in sensitivity and specificity for HPV detection while being semi-quantitative and being able to assess the viral integration status as well as detect viral variants.

Besides the above-mentioned features, our NGS assay detects several genomic regions of each included HPV type in the oncogenes E6 and E7 as well as E2 and L1. Both Cobas^®^ 4800 and CLART^®^ HPV4S, as well as several other HPV assays, detect only a region of the L1 gene [30]. However, studies have shown the possibility of L1 deletion following viral integration [38] which creates the risk of false negative HPV results in HPV assays only detecting L1 [32,55,56]. The fact that both Cobas^®^ 4800 and CLART^®^ HPV4S detect L1 poses the possibility that these two assays might be more likely to agree with each other compared to the NGS assay detecting several regions of the HPV genomes. This is a disadvantage of the comparison method used in this study where the consensus HPV results were based on HPV detection agreement between at least two of the assays. This could potentially result in a false negative consensus HPV result if both Cobas^®^ 4800 and CLART^®^ HPV4S do not detect any HPV due to L1 deletion. However, this does not seem to be the case in our cohort, which could be due to a low number of integrated cases. Since viral integration often occurs during progression of cervical dysplasia to cancer [6,7,8,9,57] and all samples analyzed in this study were ASC-US samples, the detection of a clear integration pattern in only one sample included in this study cohort was not a surprise. However, integration has been observed in several cervical cancer samples analyzed with the same NGS assay for another, yet unpublished, study in our group. Even though viral integration often results in deletion of part of the E2 gene, deletion can occur at various sites of HPV [6,38,42]. Therefore, to ensure detection of viral integration, a panel with the main purpose of assessment of the viral integration state should contain additional regions of each HPV type. The same would be the case if classification of sub-lineages should be achieved. However, this would increase both the cost and the analysis time, which may not be optimal in a screening setting. As long as the panel size is kept small, we have previously estimated a price of around 75 EUR per sample, which is within the range of PCR-based HPV analyses [32]. Despite this, a larger NGS panel could be relevant in specific HPV positive cases for further risk stratification.

As previously mentioned, because the NGS assay can detect and genotype all relevant oncogenic HPV types, it enables establishment of whether a patient has same genotype persistence or genotype switch in successive cervical samples [47]. This contrasts with the HPV assays approved for screening today where only few HPV types are genotyped. Hereby, it is not possible to confirm whether two consecutive HPV positive screening results are caused by a persistent infection or two independent infections. However, with the development of a larger NGS panel, additional variants for each HPV type could help additionally in differentiating between persistent and transient infections and thus help to avoid overtreatment of transient infections thought to be persistent.

A limitation of our study is the relatively small study cohort, which resulted in few HPV16 positive samples even after pooling the GP-collected samples and the self-samples, whereby each HPV16 positive sample had a huge impact on the calculated sensitivities for HPV16 detection. The relatively low sensitivity of 83.3% (95% CI: 58.6–96.4%) observed for CLART^®^ HPV4S was thus caused by only three HPV16 false negative samples, and the study cohort should therefore be expanded for more precise sensitivity and specificity estimations, especially regarding HPV16 detection.

Besides the aforementioned features, a strength of our NGS assay is its ability to work on both self-samples and GP-collected samples. Previous studies have shown an increase in cervical cancer screening attendance using self-sampling, and several countries have already implemented or are in the process of implementing self-sampling in their screening programs [14,25,29,58,59] which makes the ability of HPV detection in self-samples important in future HPV screening assays.

## 5. Conclusions

In conclusion, the NGS assay enables detection and genotyping of all carcinogenic, probably carcinogenic, and possibly carcinogenic HPV types while performing as well as the Cobas^®^ 4800 and CLART^®^ HPV4S assays in sensitivity and specificity for HPV detection and HPV16 detection in both GP-collected cervical samples and cervico-vaginal self-samples. The NGS assay could, thus, potentially decrease the number of false negative screening results caused by infections with HPV types not currently screened for whereby more cervical dysplasia and cervical cancer cases could be avoided. Moreover, the NGS assay gives valuable additional information about the dominating HPV type in cases with multiple HPV infections, integration status, and variants in the HPV genome which are features that could be valuable in future stratification of HPV positive women. Thus, we believe the additional information provided by NGS-based HPV analysis outweighs the longer analysis time (2–3 days) compared to PCR-based HPV analysis (hours).

## Figures and Tables

**Table 1:** HPV types detected with NGS-panel in GP-collected cervical samples and cervico-vaginal self-samples.

HPV Types	GP-Collected Cervical Samples, n (%)	Cervico-Vaginal Self-Samples, n (%)
6	1 (1.1)	5 (5.4)
16	11 (11.8)	9 (9.7)
18	3 (3.2)	2 (2.2)
30	3 (3.2)	3 (3.2)
31	8 (8.6)	5 (5.4)
33	1 (1.1)	3 (3.2)
35	4 (4.3)	6 (6.5)
39	8 (8.6)	8 (8.6)
45	3 (3.2)	6 (6.5)
51	3 (3.2)	2 (2.2)
52	5 (5.4)	4 (4.3)
53	3 (3.2)	2 (2.2)
56	1 (1.1)	0 (0.0)
58	1 (1.1)	1 (1.1)
59	4 (4.3)	2 (2.2)
66	6 (6.5)	4 (4.3)
67	2 (2.1)	5 (5.4)
68	4 (4.3)	6 (6.5)
69	1 (1.1)	1 (1.1)
70	6 (6.5)	6 (6.5)
73	2 (2.2)	3 (3.2)
82	2 (2.2)	2 (2.2)
HPV negative	46 (49.5)	49 (52.7)

GP: General practitioner.

**Table 2:** Sensitivity and specificity for detection of any ^1^ HPV type in the NGS assay, Cobas^®^ 4800, and CLART^®^ HPV4S on GP-collected cervical samples and cervico-vaginal self-samples using a consensus * as comparison.

			Consensus *			
			Positive	Negative	Sensitivity, % (95% CI)	Specificity, % (95% CI)
GP-collected cervical samples	NGS assay	Positive	42	1	97.7 (87.7–99.9)	98.0 (89.4–100.0)
		Negative	1	49		
	Cobas^®^ 4800	Positive	40	7	93.0 (80.9–98.5)	86.0 (73.3–94.2)
		Negative	3	43		
	CLART^®^ HPV4S	Positive	38	1	88.4 (74.9–96.1)	98.0 (89.4–100.0)
		Negative	5	49		
Cervico-vaginal self-samples	NGS assay	Positive	35	2	92.1 (78.6–98.3)	96.4 (87.5–99.6)
		Negative	3	53		
	Cobas^®^ 4800	Positive	36	5	94.7 (82.3–99.4)	90.9 (80.1–97.0)
		Negative	2	50		
	CLART^®^ HPV4S	Positive	37	0	97.4 (86.2–99.9)	100.0 (93.5–100.0)
		Negative	1	55		

^1^ only includes HPV types detectable in all three assays (HPV16, 18, 31, 33, 35, 39, 45, 51, 52, 56, 58, 59, 66, and 68). * Each assay was compared to the two other assays to assess a consensus result, meaning when at least two of the three assays showed the same HPV result (HPV positive or HPV negative) this result was interpreted as the consensus result. GP: General practitioner.

**Table 3:** Degree of agreement between the NGS assay and CLART^®^ HPV4S in GP-collected cervical samples and cervico-vaginal self-samples.

	GP-Collected Cervical Samples		Cervico-Vaginal Self-Samples	
Results	No. of Samples	Percentage of Samples, %	No. of Samples	Percentage of Samples, %
Agreement ^1^	75	80.7	78	83.9
Partial agreement ^2^	10	10.8	8	8.6
Disagreement ^3^	8	8.6	7	7.5

^1^ Agreement was defined as detection of the exact same HPV types in the NGS assay and CLART^®^ HPV4S. ^2^ Partial agreement was applied in samples infected with multiple HPV types and defined as detection of at least one of the same HPV types in the NGS assay and CLART^®^ HPV4S. ^3^ Disagreement was defined as detection of none of the same HPV types between the NGS assay and CLART^®^ HPV4S. GP: General practitioner.

**Table 4:** Sensitivity and specificity for detection of HPV16 in the NGS assay, Cobas^®^ 4800, and CLART^®^ HPV4S assay on GP-collected cervical samples and cervico-vaginal self-samples ^1^ using a consensus * as comparison.

		Consensus *			
		Positive	Negative	Sensitivity, % (95% CI)	Specificity, % (95% CI)
NGS assay	Positive	18	2	100.0 (81.5–100.0)	98.8 (95.8–99.9)
	Negative	0	166		
Cobas^®^ 4800	Positive	18	7	100.0 (81.5–100.0)	95.8 (91.6–98.3)
	Negative	0	161		
CLART^®^ HPV4S	Positive	15	0	83.3 (58.6–96.4)	100.0 (97.8–100.0)
	Negative	3	168		

^1^ The general practitioner-collected cervical samples and the cervico-vaginal self-samples were pooled to obtain a larger cohort. The two samples were taken independent of each other on different days and from different areas of the cervix/vagina. * Each assay was compared to the two other assays to assess a consensus result, meaning when at least two of the three assays showed the same HPV result (HPV16 positive or HPV16 negative) this result was interpreted as the consensus result. GP: General practitioner.

**Table 5:** NGS coverage data of an integrated HPV18 infection.

Amplicons	GP-Collected Sample No. 96, No. of Reads	Self-Sample No. 96, No. of Reads
BTF3	25,589	38,400
PABPN1	12,873	26,668
PPIE	13,203	14,397
RAB1B	13,495	18,686
SRSF3	18,790	26,109
HPV18_E2_1	0	0
HPV18_E2_2	0	0
HPV18_E2_3	0	0
HPV18_E6_1	1319	28
HPV18_E6_2	1868	43
HPV18_E7_1	2748	60
HPV18_E7_2	2226	35
HPV18_L1	3628	149

The upper five amplicons are included human reference genes and the eight lower amplicons cover the HPV18 genome. GP: General practitioner.

## Data Availability

Data is contained within the article.

## Supplementary Materials

The following supporting information can be downloaded at: https://www.mdpi.com/article/10.3390/cancers14030652/s1, Table S1: HPV types detected in the NGS assay, Cobas^®^ 4800, and CLART^®^ HPV4S in general practitioner-collected cervical samples and cervico-vaginal self-samples of women with atypical squamous cells of undetermined significance (ASCUS); Table S2: Genomic variants of HPVs detected by the NGS assay in general practitioner-collected cervical samples with atypical squamous cells of undetermined significance (ASCUS).

## References

[1] Walboomers J.M., Jacobs M.V., Manos M.M., Bosch F.X., Kummer J.A., Shah K.V., Snijders P.J., Peto J., Meijer C.J., Muñoz N. (1999). Human papillomavirus is a necessary cause of invasive cervical cancer worldwide. J. Pathol..

[2] The International Agency for Research on Cancer (IARC) (2007). IARC Monographs on the Evaluation of Carcinogenic Risks to Humans Volume 90 Human Papillomaviruses.

[3] Chan C.K., Aimagambetova G., Ukybassova T., Kongrtay K., Azizan A. (2019). Human Papillomavirus Infection and Cervical Cancer: Epidemiology, Screening, and Vaccination-Review of Current Perspectives. J. Oncol..

[4] Kessis T.D., Slebos R.J., Nelson W.G., Kastan M.B., Plunkett B.S., Han S.M., Lorincz A.T., Hedrick L., Cho K.R. (1993). Human papillomavirus 16 E6 expression disrupts the p53-mediated cellular response to DNA damage. Proc. Natl. Acad. Sci. USA.

[5] Gonzalez S.L., Stremlau M., He X., Basile J.R., Münger K. (2001). Degradation of the retinoblastoma tumor suppressor by the human papillomavirus type 16 E7 oncoprotein is important for functional inactivation and is separable from proteasomal degradation of E7. J. Virol..

[6] Nkili-Meyong A.A., Moussavou-Boundzanga P., Labouba I., Koumakpayi I.H., Jeannot E., Descorps-Declère S., Sastre-Garau X., Leroy E.M., Belembaogo E., Berthet N. (2019). Genome-wide profiling of human papillomavirus DNA integration in liquid-based cytology specimens from a Gabonese female population using HPV capture technology. Sci. Rep..

[7] Manawapat-Klopfer A., Wang L., Haedicke-Jarboui J., Stubenrauch F., Munk C., Thomsen L.T., Martus P., Kjaer S.K., Iftner T. (2018). HPV16 viral load and physical state measurement as a potential immediate triage strategy for HR-HPV-infected women: A study in 644 women with single HPV16 infections. Am. J. Cancer Res..

[8] Cricca M., Morselli-Labate A.M., Venturoli S., Ambretti S., Gentilomi G.A., Gallinella G., Costa S., Musiani M., Zerbini M. (2007). Viral DNA load, physical status and E2/E6 ratio as markers to grade HPV16 positive women for high-grade cervical lesions. Gynecol. Oncol..

[9] Hudelist G., Manavi M., Pischinger K.I., Watkins-Riedel T., Singer C.F., Kubista E., Czerwenka K.F. (2004). Physical state and expression of HPV DNA in benign and dysplastic cervical tissue: Different levels of viral integration are correlated with lesion grade. Gynecol. Oncol..

[10] Poljak M., Oštrbenk Valenčak A., Gimpelj Domjanič G., Xu L., Arbyn M. (2020). Commercially available molecular tests for human papillomaviruses: A global overview. Clin. Microbiol. Infect..

[11] Koliopoulos G., Nyaga V.N., Santesso N., Bryant A., Martin-Hirsch P.P., Mustafa R.A., Schünemann H., Paraskevaidis E., Arbyn M. (2017). Cytology versus HPV testing for cervical cancer screening in the general population. Cochrane Database Syst. Rev..

[12] Kurokawa T., Yoshida Y., Iwanari O., Oishi T., Kasai T., Hamada M., Fujita H., Fujiwara H., Yokoyama M., Sakuragi N. (2020). Implementation of primary HPV testing in Japan. Mol. Clin. Oncol..

[13] Machalek D.A., Roberts J.M., Garland S.M., Thurloe J., Richards A., Chambers I., Sivertsen T., Farnsworth A. (2019). Routine cervical screening by primary HPV testing: Early findings in the renewed National Cervical Screening Program. Med. J. Aust..

[14] Polman N.J., Snijders P.J.F., Kenter G.G., Berkhof J., Meijer C. (2019). HPV-based cervical screening: Rationale, expectations and future perspectives of the new Dutch screening programme. Prev. Med..

[15] Rebolj M., Rimmer J., Denton K., Tidy J., Mathews C., Ellis K., Smith J., Evans C., Giles T., Frew V. (2019). Primary cervical screening with high risk human papillomavirus testing: Observational study. BMJ.

[16] Kong T.W., Kim M., Kim Y.H., Kim Y.B., Kim J., Kim J.W., Park M.H., Park J.H., Rhee J.H., Lim M.C. (2020). High-risk human papillomavirus testing as a primary screening for cervical cancer: Position statement by the Korean Society of Obstetrics and Gynecology and the Korean Society of Gynecologic Oncology. Obs. Gynecol. Sci..

[17] Zhang J., Zhao Y., Dai Y., Dang L., Ma L., Yang C., Li Y., Kong L., Wei L., Zhang S. (2021). Effectiveness of High-risk Human Papillomavirus Testing for Cervical Cancer Screening in China: A Multicenter, Open-label, Randomized Clinical Trial. JAMA Oncol..

[18] Curry S.J., Krist A.H., Owens D.K., Barry M.J., Caughey A.B., Davidson K.W., Doubeni C.A., Epling J.W., Kemper A.R., Kubik M. (2018). Screening for Cervical Cancer: US Preventive Services Task Force Recommendation Statement. JAMA.

[19] Danske Regioners Hjemmeside (Webpage of the Danish Regions) Den Nationale Styregruppe for Livmoderhalskræftscreening (NSLS) (The National Steering Group for Cervical Cancer Screening (NSLS)). https://www.regioner.dk/nsls.

[20] Cho H.W., So K.A., Lee J.K., Hong J.H. (2015). Type-specific persistence or regression of human papillomavirus genotypes in women with cervical intraepithelial neoplasia 1: A prospective cohort study. Obs. Gynecol. Sci..

[21] Akaaboune M., Kenfack B., Viviano M., Temogne L., Catarino R., Tincho E., Mbobda J., Tran P.L., Camail R., Vassilakos P. (2018). Clearance and persistence of the human papillomavirus infection among Cameroonian women. Womens Health.

[22] National Committee for Quality Assurance Cevical Cancer Screening (CCS). https://www.ncqa.org/hedis/measures/cervical-cancer-screening/.

[23] Lynge E., Andersen B., Christensen J., Ejersbo D., Jochumsen K., Johansen T., Kristensen J.K., Larsen L.G., Mehnert F., Mikkelsen E. (2018). Cervical screening in Denmark—A success followed by stagnation. Acta Oncol..

[24] Gianino M.M., Lenzi J., Bonaudo M., Fantini M.P., Siliquini R., Ricciardi W., Damiani G. (2018). Organized screening programmes for breast and cervical cancer in 17 EU countries: Trajectories of attendance rates. BMC Public Health.

[25] Tranberg M., Bech B.H., Blaakær J., Jensen J.S., Svanholm H., Andersen B. (2018). Preventing cervical cancer using HPV self-sampling: Direct mailing of test-kits increases screening participation more than timely opt-in procedures—A randomized controlled trial. BMC Cancer.

[26] Bennett K.F., Waller J., Chorley A.J., Ferrer R.A., Haddrell J.B., Marlow L.A. (2018). Barriers to cervical screening and interest in self-sampling among women who actively decline screening. J. Med. Screen.

[27] Mangold B.R. (2019). Self-Collected Samples in Cervical Cancer Screening: Results of HPV and Pap Self-Collected Samples Compared to Physician-Obtained Specimens. Acta Cytol..

[28] Sultana F., English D.R., Simpson J.A., Brotherton J.M., Drennan K., Mullins R., Heley S., Wrede C.D., Saville M., Gertig D.M. (2014). Rationale and design of the iPap trial: A randomized controlled trial of home-based HPV self-sampling for improving participation in cervical screening by never- and under-screened women in Australia. BMC Cancer.

[29] Winer R.L., Lin J., Tiro J.A., Miglioretti D.L., Beatty T., Gao H., Kimbel K., Thayer C., Buist D.S.M. (2019). Effect of Mailed Human Papillomavirus Test Kits vs Usual Care Reminders on Cervical Cancer Screening Uptake, Precancer Detection, and Treatment: A Randomized Clinical Trial. JAMA Netw Open..

[30] De Thurah L., Bonde J., Lam J.U.H., Rebolj M. (2018). Concordant testing results between various human papillomavirus assays in primary cervical cancer screening: Systematic review. Clin. Microbiol. Infect..

[31] Nilyanimit P., Chansaenroj J., Poomipak W., Praianantathavorn K., Payungporn S., Poovorawan Y. (2018). Comparison of Four Human Papillomavirus Genotyping Methods: Next-generation Sequencing, INNO-LiPA, Electrochemical DNA Chip, and Nested-PCR. Ann. Lab. Med..

[32] Lippert J., Bønløkke S., Utke A., Knudsen B.R., Sorensen B.S., Steiniche T., Stougaard M. (2021). Targeted next generation sequencing panel for HPV genotyping in cervical cancer. Exp. Mol. Pathol..

[33] Tranberg M., Jensen J.S., Bech B.H., Blaakær J., Svanholm H., Andersen B. (2018). Good concordance of HPV detection between cervico-vaginal self-samples and general practitioner-collected samples using the Cobas 4800 HPV DNA test. BMC Infect. Dis..

[34] Tranberg M., Jensen J.S., Bech B.H., Andersen B. (2020). Urine collection in cervical cancer screening—Analytical comparison of two HPV DNA assays. BMC Infect. Dis..

[35] Gauthier J.M., Dillner J., Yaniv M. (1991). Structural analysis of the human papillomavirus type 16-E2 transactivator with antipeptide antibodies reveals a high mobility region linking the transactivation and the DNA-binding domains. Nucleic Acids Res..

[36] Kulmala S.M., Syrjänen S.M., Gyllensten U.B., Shabalova I.P., Petrovichev N., Tosi P., Syrjänen K.J., Johansson B.C. (2006). Early integration of high copy HPV16 detectable in women with normal and low grade cervical cytology and histology. J. Clin. Pathol..

[37] Arias-Pulido H., Peyton C.L., Joste N.E., Vargas H., Wheeler C.M. (2006). Human papillomavirus type 16 integration in cervical carcinoma in situ and in invasive cervical cancer. J. Clin. Microbiol..

[38] Holmes A., Lameiras S., Jeannot E., Marie Y., Castera L., Sastre-Garau X., Nicolas A. (2016). Mechanistic signatures of HPV insertions in cervical carcinomas. NPJ Genom. Med..

[39] Tsakogiannis D., Gortsilas P., Kyriakopoulou Z., Ruether I.G., Dimitriou T.G., Orfanoudakis G., Markoulatos P. (2015). Sites of disruption within E1 and E2 genes of HPV16 and association with cervical dysplasia. J. Med. Virol..

[40] Preisler S., Rebolj M., Ejegod D.M., Lynge E., Rygaard C., Bonde J. (2016). Cross-reactivity profiles of hybrid capture II, cobas, and APTIMA human papillomavirus assays: Split-sample study. BMC Cancer.

[41] De Sanjose S., Quint W.G., Alemany L., Geraets D.T., Klaustermeier J.E., Lloveras B., Tous S., Felix A., Bravo L.E., Shin H.R. (2010). Human papillomavirus genotype attribution in invasive cervical cancer: A retrospective cross-sectional worldwide study. Lancet Oncol..

[42] Hu Z., Zhu D., Wang W., Li W., Jia W., Zeng X., Ding W., Yu L., Wang X., Wang L. (2015). Genome-wide profiling of HPV integration in cervical cancer identifies clustered genomic hot spots and a potential microhomology-mediated integration mechanism. Nat. Genet..

[43] Halec G., Alemany L., Lloveras B., Schmitt M., Alejo M., Bosch F.X., Tous S., Klaustermeier J.E., Guimerà N., Grabe N. (2014). Pathogenic role of the eight probably/possibly carcinogenic HPV types 26, 53, 66, 67, 68, 70, 73 and 82 in cervical cancer. J. Pathol..

[44] Petry K.U., Liebrich C., Luyten A., Zander M., Iftner T. (2017). Surgical staging identified false HPV-negative cases in a large series of invasive cervical cancers. Papillomavirus Res..

[45] Amaro-Filho S.M., Gradissimo A., Usyk M., Moreira F.C.B., de Almeida L.M., Moreira M.A.M., Burk R.D. (2020). HPV73 a nonvaccine type causes cervical cancer. Int. J. Cancer.

[46] Salazar K.L., Duhon D.J., Olsen R., Thrall M. (2019). A review of the FDA-approved molecular testing platforms for human papillomavirus. J. Am. Soc. Cytopathol..

[47] Bonde J., Bottari F., Iacobone A.D., Cocuzza C.E., Sandri M.T., Bogliatto F., Khan K.S., Ejegod D.M., Gary D.S., Andrews J.C. (2021). Human Papillomavirus Same Genotype Persistence and Risk: A Systematic Review. J. Low Genit Tract Dis..

[48] Thomsen L.T., Kjaer S.K., Munk C., Ørnskov D., Waldstrøm M. (2021). Benefits and potential harms of human papillomavirus (HPV)-based cervical cancer screening: A real-world comparison of HPV testing versus cytology. Acta Obs. Gynecol. Scand..

[49] Lu X., Wang T., Zhang Y., Liu Y. (2021). Analysis of influencing factors of viral load in patients with high-risk human papillomavirus. Virol. J..

[50] Burk R.D., Harari A., Chen Z. (2013). Human papillomavirus genome variants. Virology.

[51] Mirabello L., Yeager M., Cullen M., Boland J.F., Chen Z., Wentzensen N., Zhang X., Yu K., Yang Q., Mitchell J. (2016). HPV16 Sublineage Associations With Histology-Specific Cancer Risk Using HPV Whole-Genome Sequences in 3200 Women. J. Natl. Cancer Inst..

[52] Tan G., Duan M., Li Y., Zhang N., Zhang W., Li B., Qu P. (2019). Distribution of HPV 16 E6 gene variants in screening women and its associations with cervical lesions progression. Virus Res..

[53] Mirabello L., Yeager M., Yu K., Clifford G.M., Xiao Y., Zhu B., Cullen M., Boland J.F., Wentzensen N., Nelson C.W. (2017). HPV16 E7 Genetic Conservation Is Critical to Carcinogenesis. Cell.

[54] Zhang L., Liao H., Yang B., Geffre C.P., Zhang A., Zhou A., Cao H., Wang J., Zhang Z., Zheng W. (2015). Variants of human papillomavirus type 16 predispose toward persistent infection. Int. J. Clin. Exp. Pathol..

[55] Lagheden C., Eklund C., Lamin H., Kleppe S.N., Lei J., Elfström K.M., Sundström K., Andrae B., Sparén P., Dillner J. (2018). Nationwide comprehensive human papillomavirus (HPV) genotyping of invasive cervical cancer. Br. J. Cancer.

[56] Molijn A., Jenkins D., Chen W., Zhang X., Pirog E., Enqi W., Liu B., Schmidt J., Cui J., Qiao Y. (2016). The complex relationship between human papillomavirus and cervical adenocarcinoma. Int. J. Cancer.

[57] Gimenes F., Souza R.P., de Abreu A.L., Pereira M.W., Consolaro M.E., da Silva V.R. (2016). Simultaneous detection of human papillomavirus integration and c-MYC gene amplification in cervical lesions: An emerging marker for the risk to progression. Arch. Gynecol. Obs..

[58] Sultana F., English D.R., Simpson J.A., Drennan K.T., Mullins R., Brotherton J.M., Wrede C.D., Heley S., Saville M., Gertig D.M. (2016). Home-based HPV self-sampling improves participation by never-screened and under-screened women: Results from a large randomized trial (iPap) in Australia. Int. J. Cancer.

[59] Saville M., Hawkes D., McLachlan E., Anderson S., Arabena K. (2018). Self-collection for under-screened women in a National Cervical Screening Program: Pilot study. Curr. Oncol..

