# Supplementary material for: Targeted Next Generation Sequencing for Human Papillomavirus Genotyping in Cervical Liquid-Based Cytology Samples
Source: Cancers (Basel). 2022 Jan 27;14(3):652. doi: 10.3390/cancers14030652 (PMC8833452; doi:10.3390/cancers14030652)
Supplement: Supplementary file 1 [file cancers-14-00652-s001.zip › cancers-1557536-supplementary.pdf]

Supplementary tables

Table S1: HPV types detected in the NGS assay, Cobas® 4800, and CLART® HPV4S in general practitioner-collected cervical samples and cervico-vaginal self-samples of women with atypical squamous cells of undetermined significance (ASCUS).

	GP-collected cervical samples			Cervico-vaginal self-samples		
**Patient no.**	**NGS assay**	**Cobas® 4800**	**CLART® HPV4S**	**NGS assay**	**Cobas® 4800**	**CLART® HPV4S**
**7**	HPVneg	HPVneg	HPVneg	HPVneg	HPVneg	HPVneg
**8**	HPVneg	HPVneg	HPVneg	HPV68	HPVneg	HPV68
**9**	HPVneg	HPVneg	HPV68	HPV68	HPVneg	HPVneg
**10**	HPV16<br>HPV66	HPV16<br>HPVA	HPV16<br>HPV66	HPV16<br>HPV66	HPV16<br>HPVA	HPV66
**11**	HPVneg	HPVneg	HPVneg	HPVneg	HPVneg	HPVneg
**12**	HPVneg	HPVneg	HPVneg	HPVneg	HPVneg	HPVneg
**13**	HPV16<br>HPV30<br>HPV31<br>HPV66<br>HPV68	HPV16<br>HPVA	HPV31<br>HPV66	HPV6<br>HPV30<br>HPV31<br>HPV66<br>HPV67<br>HPV68	HPV16<br>HPVA	HPV6<br>HPV31<br>HPV66<br>HPV68
**16**	HPVneg	HPVneg	HPVneg	HPVneg	HPVneg	HPVneg
**17**	HPVneg	HPVneg	HPVneg	HPVneg	HPVneg	HPVneg
**18**	HPVneg	HPVA	HPVneg	HPVneg	HPVneg	HPVneg
**19**	HPVneg	HPVneg	HPVneg	HPVneg	HPVneg	HPVneg
**21**	HPV58<br>HPV67	HPVA	HPV58	HPV67	HPVneg	HPVneg
**23**	HPVneg	HPVneg	HPVneg	HPV35	HPVneg	HPVneg
**26**	HPVneg	HPVneg	HPVneg	HPV6	HPVneg	HPVneg
**27**	HPVneg	HPVneg	HPVneg	HPVneg	HPVneg	HPVneg
**28**	HPV45<br>HPV73	HPVA	HPV45	HPVneg	HPVA	HPVneg
**29**	HPVneg	HPVneg	HPVneg	HPVneg	HPVneg	HPVneg
**31**	HPV31<br>HPV33<br>HPV66	HPVA	HPV33	HPV33	HPVA	HPV33
**32**	HPVneg	HPVneg	HPVneg	HPVneg	HPVneg	HPVneg
**34**	HPV39	HPVA	HPV39	HPV39	HPVA	HPV39
**35**	HPV56	HPVA	HPV56	HPVneg	HPVA	HPVneg
**37**	HPVneg	HPVneg	HPVneg	HPVneg	HPVA	HPVneg
**38**	HPV39	HPVA	HPV39	HPV35<br>HPV39<br>HPV68<br>HPV73	HPVA	HPV35<br>HPV39<br>HPV68
**39**	HPVneg	HPVneg	HPVneg	HPVneg	HPVneg	HPVneg

**40**	HPV30<br>HPV68<br>HPV73	HPVneg	HPV68	HPV68<br>HPV73	HPVneg	HPV68
**41**	HPV16<br>HPV66	HPVneg	HPVneg	HPVneg	HPVneg	HPVneg
**44**	HPV51<br>HPV82	HPVA	HPV51	HPV6<br>HPV82	HPVA	HPV6
**45**	HPVneg	HPVneg	HPVneg	HPVneg	HPVneg	HPVneg
**46**	HPV31<br>HPV39	HPVA	HPV31<br>HPV39	HPV31<br>HPV33<br>HPV39	HPVA	HPV31<br>HPV39
**47**	HPV39	HPVA	HPV39	HPV39	HPVA	HPV39
**49**	HPV52<br>HPV59<br>HPV69	HPVA	HPV59	HPV45<br>HPV52<br>HPV59<br>HPV66<br>HPV69	HPVA	HPV51<br>HPV59
**50**	HPV16<br>HPV45<br>HPV51	HPV16<br>HPVA	HPV16<br>HPV51	HPV45<br>HPV51<br>HPV67	HPV16<br>HPVA	HPV51
**51**	HPVneg	HPVA	HPVneg	HPVneg	HPVneg	HPVneg
**52**	HPV39<br>HPV82	HPVA	HPV39<br>HPV68	HPV39<br>HPV82	HPVA	HPV39<br>HPV68
**55**	HPV39	HPVA	HPV39	HPV39	HPV16<br>HPVA	HPV39
**56**	HPVneg	HPVneg	HPVneg	HPVneg	HPVneg	HPVneg
**57**	HPV18	HPV18	HPVneg	HPVneg	HPVneg	HPVneg
**59**	HPV35	HPVA	HPV35	HPVneg	HPVneg	HPVneg
**60**	HPVneg	HPVneg	HPVneg	HPVneg	HPVneg	HPVneg
**61**	HPV6	HPVneg	HPV6	HPV6	HPVneg	HPV6
**62**	HPVneg	HPVneg	HPVneg	HPVneg	HPVneg	HPVneg
**64**	HPV53	HPVneg	HPVneg	HPVneg	HPVneg	HPVneg
**65**	HPV31<br>HPV70	HPVA	HPV31	HPV31	HPVA	HPV31
**66**	HPVneg	HPVneg	HPVneg	HPVneg	HPVneg	HPVneg
**67**	HPVneg	HPVneg	HPVneg	HPVneg	HPVneg	HPVneg
**71**	HPVneg	HPVneg	HPVneg	HPVneg	HPVneg	HPVneg
**72**	HPVneg	HPVneg	HPVneg	HPVneg	HPVneg	HPVneg
**73**	HPVneg	HPVneg	HPVneg	HPVneg	HPVneg	HPVneg
**76**	HPV16	HPV16<br>HPVA	HPV16<br>HPV51	HPV16	HPV16	HPV16
**80**	HPVneg	HPVneg	HPVneg	HPVneg	HPVneg	HPVneg
**83**	HPV16<br>HPV70	HPV16	HPVneg	HPV16<br>HPV70	HPV16	HPV16

**84**	HPV18<br>HPV35	HPV18<br>HPVA	HPV18<br>HPV35	HPV18<br>HPV35	HPV18<br>HPVA	HPV18<br>HPV35
**87**	HPV59	HPVA	HPVneg	HPV59<br>HPV67	HPVA	HPV59
**92**	HPV67<br>HPV70	HPVA	HPVneg	HPV66<br>HPV67<br>HPV70	HPVA	HPV66
**96**	HPV18<br>HPV31	HPV18	HPV18	HPV18	HPV18	HPV18
**99**	HPV39	HPVA	HPV39	HPV39	HPVA	HPV39
**101**	HPVneg	HPVneg	HPVneg	HPVneg	HPVneg	HPVneg
**104**	HPV16<br>HPV68	HPV16	HPV16<br>HPV68	HPV16<br>HPV68	HPV16	HPV16<br>HPV68
**107**	HPV66<br>HPV70	HPVA	HPV66	HPV70	HPVneg	HPVneg
**123**	HPV59	HPV16<br>HPVA	HPVneg	HPV16	HPV16<br>HPVA	HPV16<br>HPV59
**128**	HPVneg	HPVneg	HPVneg	HPVneg	HPVneg	HPVneg
**130**	HPV53<br>HPV68	HPVA	HPV68	HPV53	HPVneg	HPVneg
**131**	HPVneg	HPVneg	HPVneg	HPVneg	HPVneg	HPVneg
**135**	HPVneg	HPVneg	HPVneg	HPVneg	HPVA	HPV51
**137**	HPVneg	HPVneg	HPVneg	HPVneg	HPVneg	HPVneg
**138**	HPV16<br>HPV30<br>HPV53	HPV16	HPV16	HPV16<br>HPV30<br>HPV45<br>HPV53	HPV16<br>HPVA	HPV16<br>HPV45
**139**	HPVneg	HPV18	HPVneg	HPVneg	HPV18<br>HPVA	HPV66
**144**	HPVneg	HPVneg	HPVneg	HPVneg	HPVneg	HPVneg
**145**	HPVneg	HPVneg	HPVneg	HPVneg	HPVneg	HPVneg
**147**	HPV16	HPV16	HPV16	HPV16	HPV16	HPV16
**148**	HPV52<br>HPV59<br>HPV66	HPVneg	HPV51<br>HPV52<br>HPV59	HPVneg	HPVneg	HPVneg
**149**	HPV16	HPVA	HPVneg	HPVneg	HPV18<br>HPVA	HPV35
**153**	HPV35<br>HPV70	HPV18<br>HPVA	HPV35	HPV35<br>HPV70	HPVA	HPV35
**156**	HPV31<br>HPV39<br>HPV51	HPVA	HPV31<br>HPV39<br>HPV51<br>HPV58	HPV31<br>HPV39<br>HPV51<br>HPV58<br>HPV68	HPVA	HPV31<br>HPV39<br>HPV51<br>HPV58

**163**	HPV52	HPVA	HPV52	HPV6<br>HPV52	HPVA	HPV6<br>HPV52
**165**	HPVneg	HPVA	HPVneg	HPV45<br>HPV73	HPV18<br>HPVA	HPVneg
**168**	HPVneg	HPV16<br>HPVA	HPVneg	HPVneg	HPV16	HPVneg
**169**	HPVneg	HPVneg	HPVneg	HPVneg	HPVneg	HPVneg
**179**	HPV35	HPVA	HPV35	HPV35	HPVA	HPV35
**180**	HPVneg	HPVneg	HPVneg	HPVneg	HPVneg	HPVneg
**184**	HPVneg	HPVneg	HPVneg	HPVneg	HPVneg	HPVneg
**185**	HPVneg	HPVA	HPV68	HPVneg	HPVneg	HPVneg
**189**	HPVneg	HPVneg	HPVneg	HPV16<br>HPV35<br>HPV70	HPV16<br>HPVA	HPV16<br>HPV35
**190**	HPV31	HPVneg	HPV31	HPVneg	HPVneg	HPVneg
**191**	HPVneg	HPVneg	HPVneg	HPVneg	HPVneg	HPVneg
**192**	HPVneg	HPVneg	HPVneg	HPVneg	HPVneg	HPVneg
**193**	HPVneg	HPVneg	HPVneg	HPVneg	HPVneg	HPVneg
**195**	HPV52	HPVA	HPV52	HPV52	HPVA	HPV52
**196**	HPV31	HPVA	HPV31	HPV31<br>HPV33	HPVA	HPV31
**202**	HPVneg	HPVneg	HPVneg	HPVneg	HPVneg	HPVneg
**205**	HPV45<br>HPV52	HPVA	HPV45<br>HPV52	HPV45<br>HPV52	HPVA	HPV45<br>HPV52
**211**	HPV70	HPV16	HPVneg	HPV30<br>HPV70	HPVneg	HPVneg
**212**	HPV16	HPV16<br>HPVA	HPV16<br>HPV45	HPV16<br>HPV45	HPV16<br>HPVA	HPV16<br>HPV45

HPVA: HPV31, 33, 35, 39, 45, 51, 52, 56, 58, 59, 66, or 68, HPVneg: HPV negative, GP: General practitioner.

Table S2: Genomic variants of HPVs detected by the NGS assay in general practitioner-collected cervical samples with atypical squamous cells of undetermined significance (ASCUS).

HPV type	Genomic variant	Amino acid variant	Viral gene	Frequency, n (%)
**6**	c.221A>T	p.Thr40Thr	E6	1 (100.00)
	c.365A>T	p.Thr88Thr	E6	1 (100.00)
	c.392C>T	p.Ile97Ile	E6	1 (100.00)
	c.823C>A	p.Thr98Thr	E7	1 (100.00)
	c.3387T>C	p.Leu222Pro<br>p.Leu27Leu	E2<br>E4	1 (100.00)
	c.3404A>C	p.Lys228Gln<br>p.Pro32Pro	E2<br>E4	1 (100.00)
	c.3514C>G	p.His264Gln<br>p.Thr69Arg	E2<br>E4	1 (100.00)
	c.3518C>G	p.Leu266Val<br>p.Thr70Thr	E2<br>E4	1 (100.00)
	c.3540A>C	p.Gln273Pro<br>p.Ser78Arg	E2<br>E4	1 (100.00)
	c.3551C>A	p.Arg277Arg<br>p.Asp81Glu	E2<br>E4	1 (100.00)
	c.6721A>G	p.Lys311Lys	L1	1 (100.00)
**16**	c.3365G>A	p.Glu204Lys<br>p.Thr8Thr	E2<br>E4	2 (18.18)
	c.3384T>C	p.Ile210Thr<br>p.Leu15Leu	E2<br>E4	1 (9.09)
	c.3410C>T	p.Pro219Ser<br>p.Thr23Thr	E2<br>E4	7 (63.64)
	c.3485G>A	p.Glu244Lys<br>p.Gln48Gln	E2<br>E4	1 (9.09)
	c.6568T>G	p.Pro310Pro	L1	1 (9.09)
**18**	c.751C>T	p.Ala54Ala	E7	3 (100.00)
	c.3630C>G	p.Pro272Ala<br>p.His71Gln	E2<br>E4	2 (66.67)
**30**	c.400T>C	p.Phe100Ser	E6	2 (66.67)
	c.433A>T	p.Gln111Leu	E6	1 (33.33)
	c.800C>T	p.Pro79Ser	E7	3 (100.00)
	c.808G>A	p.Lys81Lys	E7	3 (100.00)
	c.3464T>C	p.Pro246Pro<br>p.Leu51Pro	E2<br>E4	3 (100.00)
	c.3549A>T	p.Thr275Ser<br>p.Thr79Thr	E2<br>E4	1 (33.33)
	c.3602C>T	p.Asp292Asp<br>p.Thr97Met	E2<br>E4	3 (100.00)
	c.3614A>G	p.Lys296Lys<br>p.Lys101Arg	E2<br>E4	3 (100.00)

	c.6605A>G	p.Gly325Gly	L1	1 (33.33)
	c.6620T>A	p.Ile330Ile	L1	2 (66.67)
**31**	c.248T>C	p.Phe47Phe	E6	3 (37.50)
	c.404G>A	p.Leu99Leu	E6	3 (37.50)
	c.428A>G	p.Gln107Gln	E6	3 (37.50)
	c.580G>A	p.Thr7Thr	E7	3 (37.50)
	c.626C>T	p.His23Tyr	E7	5 (62.50)
	c.743A>G	p.Lys62Glu	E7	8 (100.00)
	c.3517C>T	p.Ile275Ile<br>p.Ser80Leu	E2<br>E4	1 (12.50)
**33**	c.213A>C	p.Lys35Asn	E6	1 (100.00)
	c.2877G>T	p.Leu43Phe	E2	1 (100.00)
	c.3401A>C	p.Asn218Thr<br>p.Thr23Pro	E2<br>E4	1 (100.00)
**35**	c.3465A>G	p.Tyr250Cys<br>p.Thr55Ala	E2<br>E4	4 (100.00)
**39**	c.3485T>C	p.Ser230Pro<br>p.Ile28Ile	E2<br>E4	2 (25.00)
	c.3567C>G	p.Pro257Arg<br>p.Pro56Ala	E2<br>E4	1 (12.50)
	c.3621A>C	p.Asn275Thr<br>p.Thr74Pro	E2<br>E4	1 (12.50)
	c.3623_3625del	p.Ser276del<br>p.Val75del	E2<br>E4	1 (12.50)
	c.3631_3634delinsT	p.Gly278_His279delinsGly<br>p.Ala77_Thr78delinsVal	E2<br>E4	1 (12.50)
	c.3636A>C	p.Asn280Thr<br>p.Thr79Pro	E2<br>E4	1 (12.50)
	c.3640A>C	p.Thr281Thr<br>p.Gln80Pro	E2<br>E4	2 (25.00)
	c.6569T>G	p.Pro309Pro	L1	1 (12.50)
	c.6638T>A	p.Val332Val	L1	6 (75.00)
**45**	c.808G>T	p.Glu74Asp	E7	2 (66.67)
	c.2898T>G	p.Leu44Val	E2	2 (66.67)
	c.2899T>G	p.Leu44Trp	E2	1 (33.33)
	c.3510A>G	p.Thr248Ala<br>p.Leu45Leu	E2<br>E4	2 (66.67)
	c.3521T>C	p.Pro251Pro<br>p.Leu49Pro	E2<br>E4	2 (66.67)
	c.6615A>G	p.Val362Val	L1	1 (33.33)
**51**	c.2874G>A	p.Arg52Gln	E2	2 (66.67)
**52**	c.375G>T	p.Val92Leu	E6	1 (20.00)
	c.404T>C	p.Ile101Ile	E6	1 (20.00)

	c.3409G>T	p.Val223Leu<br>p.Gln27His	E2<br>E4	2 (40.00)
	c.3542G>A	p.Arg267Gln<br>p.Gly72Arg	E2<br>E4	1 (20.00)
**53**	c.224G>A	p.Leu41Leu	E6	1 (33.33)
	c.230G>T	p.Ala43Ala	E6	1 (33.33)
	c.261C>T	p.Leu54Leu	E6	1 (33.33)
	c.412C>T	p.Ser104Leu	E6	1 (33.33)
	c.803T>C	p.Ser79Pro	E7	1 (33.33)
	c.3434T>A	p.Tyr231Asn<br>p.Pro35Pro	E2<br>E4	1 (33.33)
	c.3471C>T	p.Pro243Leu<br>p.Pro48Ser	E2<br>E4	1 (33.33)
	c.3542G>C	p.Glu267Gln<br>p.Gln71His	E2<br>E4	2 (66.67)
	c.3565C>T	p.Asp274Asp<br>p.Thr79Ile	E2<br>E4	1 (33.33)
	c.6602G>A	p.Gln315Gln	L1	1 (33.33)
**56**	c.141A>C	p.Ser14Arg	E6	1 (100.00)
	c.802G>C	p.Gln77His	E7	1 (100.00)
	c.3383G>C	p.Val213Leu<br>p.Leu17Leu	E2<br>E4	1 (100.00)
	c.3499T>C	p.Phe251Phe<br>p.Leu56Ser	E2<br>E4	1 (100.00)
	c.3545C>A	p.His267Asn<br>p.His71Gln	E2<br>E4	1 (100.00)
**58**	c.2935A>C	p.Ser61Ser	E2	1 (100.00)
	c.6641G>A	p.Val333Val	L1	1 (100.00)
**59**	c.213T>C	p.Phe53Phe	E6	1 (25.00)
	c.402T>C	p.Asp116Asp	E6	3 (75.00)
	c.595A>C	p.Gln18His	E7	3 (75.00)
	c.600A>T	p.Tyr20Phe	E7	3 (75.00)
	c.754T>C	p.Asn71Asn	E7	1 (25.00)
	c.3313A>T	p.Tyr193Phe	E2	1 (25.00)
	c.3378G>T	p.Ala215Ser<br>p.Leu14Leu	E2<br>E4	4 (100.00)
**66**	c.539G>C	p.Thr146Thr	E6	2 (33.33)
	c.551T>C	p.Ala150Ala	E6	6 (100.00)
	c.602G>A	p.Val11Ile	E7	6 (100.00)
	c.628G>T	p.Thr19Thr	E7	6 (100.00)
	c.779_781delinsCTT	p.Leu70Leu	E7	6 (100.00)
	c.817G>A	p.Glu82Glu	E7	1 (16.67)
	c.3411T>C	p.Phe228Ser<br>p.Leu33Leu	E2<br>E4	3 (50.00)

	c.3433G>A	p.Ala235Ala<br>p.Arg40Gln	E2<br>E4	1 (16.67)
	c.3557C>G	p.Pro277Ala<br>p.Val81Val	E2<br>E4	4 (66.67)
	c.3574A>C	p.Gln282His<br>p.Lys87Thr	E2<br>E4	4 (66.67)
	c.3576G>A	p.Ser283Asn<br>p.Ala88Thr	E2<br>E4	4 (66.67)
	c.3592A>G	p.Lys288Lys<br>p.Lys93Arg	E2<br>E4	4 (66.67)
	c.3598G>A	p.Thr290Thr<br>p.Arg95His	E2<br>E4	4 (66.67)
	c.3607C>T	p.Ile293Ile	E2	4 (66.67)
**67**	c.252_254delinsCAC	p.Lys51His	E6	1 (50.00)
	c.405C>A	p.Arg102Arg	E6	2 (100.00)
	c.648A>T	p.His29Leu	E7	1 (50.00)
	c.775T>C	p.Ile71Ile	E7	1 (50.00)
	c.2865C>A	p.Leu49Met	E2	2 (100.00)
	c.2879G>A	p.Arg53Arg	E2	1 (50.00)
	c.6611T>C	p.Val332Val	L1	1 (50.00)
**68**	c.148A>G	p.Ser50Gly	E6	1 (25.00)
	c.154C>T	p.Leu52Leu	E6	1 (25.00)
	c.157_158delinsAA	p.Cys53Asn	E6	1 (25.00)
	c.536A>G	p.Tyr18Cys	E7	1 (25.00)
	c.547C>G	p.Gln22Glu	E7	1 (25.00)
	c.2866G>T	p.Pro60Pro	E2	1 (25.00)
	c.2902T>C	p.Ala72Ala	E2	1 (25.00)
	c.2930C>A	p.Leu82Ile	E2	1 (25.00)
	c.2936A>C	p.Lys84Gln	E2	1 (25.00)
	c.6483C>T	p.Thr332Thr	L1	1 (25.00)
**69**	c.677G>T	p.Glu38Asp	E7	1 (100.00)
	c.793T>G	p.Leu77Arg	E7	1 (100.00)
**70**	c.404A>G	p.Asn100Asp	E6	6 (100.00)
	c.3503G>A	p.Ala231Thr<br>p.Pro29Pro	E2<br>E4	1 (16.67)
	c.3516C>T	p.Pro235Leu<br>p.His34Tyr	E2<br>E4	1 (16.67)
	c.3568A>G	p.Arg252Arg<br>p.Asp51Gly	E2<br>E4	1 (16.67)
**73**	c.203C>T	p.Cys34Cys	E6	1 (50.00)
	c.372_374delinsCAA	p.Asn91Gln	E6	1 (50.00)
	c.394G>A	p.Cys98Tyr	E6	1 (50.00)
	c.396A>G	p.Asn99Asp	E6	1 (50.00)
	c.641A>G	p.Asn31Ser	E7	1 (50.00)
	c.789G>T	p.Glu80Asp	E7	2 (100.00)

	c.3412C>T	p.Thr224Thr<br>p.Pro27Lleu	E2<br>E4	2 (100.00)
	c.3470T>C	p.Ser244Pro<br>p.Thr46Thr	E2<br>E4	2 (100.00)
	c.3490_3492del	p.Cys251del<br>p.Val54del	E2<br>E4	1 (50.00)
**82**	c.744C>G	p.His60Gln	E7	2 (100.00)
	c.810T>G	p.Phe82Leu	E7	1 (50.00)
	c.2937A>C	p.Val64Val	E2	1 (50.00)
	c.3315T>C	p.Asn190Asn	E2	1 (50.00)
	c.3356C>A	p.Thr204Asn<br>p.Pro9Thr	E2<br>E4	2 (100.00)
	c.3553G>T	p.Gly270Cys<br>p.Gln74His	E2<br>E4	2 (100.00)
	c.3564C>T	p.Thr273Thr<br>p.Pro78Leu	E2<br>E4	2 (100.00)
	c.6560A>G	p.Ile331Val	L1	1 (50.00)
	c.6582A>G	p.Lys338Arg	L1	2 (100.00)
